# Dataset on managerial incentives and bank performance: Evidence from Nigerian deposit money banks

**DOI:** 10.1016/j.dib.2018.05.093

**Published:** 2018-05-25

**Authors:** Olorunfemi Adebisi Onakoya, Chinonye Love Moses, Maxwell Ayodele Olokundun, Uchechukwu Emena Okorie, Fatai Alani Lawal

**Affiliations:** a Department of Business Management, Covenant University, Ota, Ogun State, Nigeria; b Department of Economics, Covenant University, Ota, Ogun State, Nigeria

**Keywords:** CEO remuneration, Managerial incentives, Nigerian deposit money banks, Performance, Corporate governance, Board chairman pay

## Abstract

This study presents a data-set on the influence of managerial incentives on bank performance in selected Deposit Money Banks (DMB) in Nigeria. The use of managerial incentives to align interests of the principal and agent is advocated by agency theorists, but the peculiarity of the banking industry in terms of the role of regulation, capital structure, opacity and complexity of its transactions among others presents a different proposition in corporate governance research. The data collected over a longitudinal period between 2006 and 2016, provide information on specific managerial incentives and financial performance measures. Descriptive and inferential statistics such as correlation, and panel regression analysis estimates are presented. When analysed, the data can be a pointer in determining the unique managerial incentives predictors that could enhance a bank׳s performance.

**Specifications Table**

**Table:** 

Subject area	*Business Management*
More specific subject area	*Corporate Governance*
Type of data	*Tables and figures*
How data was acquired	*Secondary data (Manually extracted from banks’ annual reports)*
Data format	*Raw, analysed*
Experimental factors	*Sample consists of eight deposit money banks in Nigeria*
Experimental features	*Descriptive statistics, and panel data regression*
Data source location	*Lagos, Nigeria*
Data accessibility	*Data included in this article*

**Value of the data**

• Data was manually extracted from selected banks׳ annual reports, and stock market reports. It comprises the most expansive and currently available data since the Nigerian banking consolidation exercise (2006–2016).
• Dataset on managerial incentives can be used to explore other research interests – such as Executive Pay and Banking Risk, CEO-employee pay ratio, Determinants of CEO pay, CEO pay and Environmental Performance etc.
• Sourcing data on managerial incentives, and historical stock market prices for research is quite challenging in a developing country like Nigeria, hence scientific conclusions can be drawn from the dataset.
• The dataset can be used by academia, managers, board, investors, and regulators to identify specific managerial incentives as predictors of bank performance.

## 1 Data

The dataset contains raw descriptive and inferential statistics. Panel data regression analysis was used to test the relationships between managerial incentives and bank performance. Table 1 provides data about the descriptive statistics for the study variables. Table 2 provides data on correlations and variance inflation factors for the variables used in the empirical analysis, while Table 3 provides data on the estimates of the panel regression specification.

**Table 1:** Descriptive statistics – managerial incentives and bank performance.

	CEOR	CEOT	BCREM	BCT	CSO	BGDR	ROA	ROE	TBQ	NIM
	*NGN ׳m*	*Years*	*NGN ׳m*	*Years*	*No. (‘m)*	*%*	*%*	*%*	*No.*	*%*
Mean	63.80	4.43	14.88	3.70	267.00	11.67	2.13	14.73	1.34	13.99
Median	65.40	3.50	8.41	3.00	73.87	12.92	2.35	14.72	0.96	13.58
Maximum	205.00	19.00	201.00	11.00	2,690.00	33.33	5.94	36.56	5.77	32.39
Minimum	6.50	1.00	0.40	1.00	0.004	–	(2.26)	(20.89)	0.09	5.81
Std. Dev.	45.50	3.68	23.15	2.68	524.00	10.63	1.52	11.05	1.30	4.13

NGN –Nigerian Naira, ‘m – millions.

**Table 2:** Correlation – managerial incentives and bank performance.

Variables	1	2	3	4	5	6	7	8	9	10
1. lnBGDR	1									
2. lnBCT	−0.03	1								
3. lnBCREM	0.09	0.24*	1							
4. lnCEOR	0.36***	0.11	0.43***	1						
5. lnCSO	0.33**	0.06	−0.02	0.02	1					
6. lnCEOT	−0.22	0.16	−0.12	−0.06	0.24*	1				
7. lnNIM	−0.29**	−0.19	−0.29**	−0.54***	−0.16	0.2	1			
8. lnROA	−0.02	−0.25*	−0.12	0.17	0.06	0.22	0.24	1		
9. lnROE	0.04	−0.31**	−0.25*	0.08	0.07	0.14	0.31**	0.93***	1	
10. lnTBQ	−0.2	−0.41***	−0.34**	−0.28**	−0.23*	0.11	0.63***	0.59***	0.64***	1
V.I.F	**1.44**	**1.12**	**1.33**	**1.43**	**1.26**	**1.26**				

***, **, and * denote significance at the 0.01, 0.05, and 0.10 levels, respectively.

**Table 3:** Panel regression – managerial incentives and bank performance.

Variable	Description	ROA	ROE	TBQ	NIM
C	Constant	4.547	7.646	16.249	6.682
		(−2.21)	(3.408)	(8.039)	(8.854)
LCEOR	CEO remuneration	0.134	0.084	−0.436***	−0.206***
		(−1.26)	(0.720)	(−4.147)	(−5.240)
LCEOT	CEO Tenure	0.181	0.111	0.058	0.069
		(−1.68)	(0.945)	(0.546)	(1.742)
LBGDR	Board Gender	0.040	0.055	0.111	0.054
		(−0.18)	(0.232)	(0.583)	(0.767)
LBCT	Board Chair Tenure	0.001	−0.016	0.036	0.108**
		(−0.01)	(−0.122)	(0.315)	(2.530)
LCSO	CEO Share Ownership	−0.074	−0.081	−0.192***	−0.001
		(−1.24)	(−1.252)	(−3.321)	(−0.067)
LBCREM	Board Chair Remuneration	−0.326***	−0.328**	−0.349***	−0.045
		(−2.74)	(−2.525)	(−3.077)	(−1.052)
R^2^		0.675	0.612	0.796	0.695
Adjusted R^2^		0.580	0.499	0.739	0.610
F-Stat		7.087	5.398	13.988	8.185
Prob (F-Stat)		0.000	0.000	0.000	0.000

***, **, and * denote significance at the 0.01, 0.05, and 0.10 levels, respectively. The *t*-statistics (reported in parenthesis).

## 2 Experimental design, materials and methods

Data for the study on managerial incentives were manually extracted from the annual reports (directors’ profile, and notes to the accounts) of the selected banks, while performance measures were computed based on the banks’ financials and online stock market reports. The eight (8) deposit money banks selected for the study were the final sample after meeting the evaluation criteria which included among others the availability of data for the period under study. The banks are from both Tier 1 and Tier 2 categories, while five (5) of the banks control seventy percent (70%) of the market share. The study identified six (6) components of managerial incentives from literature provided by Refs. [1], [2], [5], [6], [7], [8]. These are: CEOs Remuneration (CEOR), CEOs Tenure (CEOT), Board Chairman׳s Remuneration (BCREM), Board Chairman׳s Tenure (BCT), CEO׳s Share Ownership (CSO), and Board Gender (BGDR). Four proxies of bank performance adopted include: Return on Assets (ROA), Return on Equity (ROE), Tobin׳s Q (TBQ), and Net Interest Margin (NIM) as used in Refs. [8], [9], [10]. A panel data regression comprising consistent fixed effect estimates, efficient random effect estimates and Hausman test was adopted to analyse the dataset. This is consistent with studies in Refs. [3], [4], [5]. Preliminary tests on analysis of measures involving correlation matrix, variance inflation factors to test multi-collinearity was performed. The data was analysed using the Eviews software Version 9. Econometric analysis was adopted to determine the nature of the relationship between managerial incentives and bank performance using the following panel regression model:

$${\mathrm{BPerf}}_{\mathrm{it}}=\beta 0_{\mathrm{it}}+\beta 1{\mathrm{lnCEOR}}_{\mathrm{it}}+\beta 2{\mathrm{lnCEOT}}_{\mathrm{it}}+\beta 3{\mathrm{lnBCREM}}_{\mathrm{it}}+\beta 4{\mathrm{lnBCT}}_{\mathrm{it}}+\beta 5{\mathrm{lnCSO}}_{\mathrm{it}}+\beta 6{\mathrm{lnBGDR}}_{\mathrm{it}}+U_{\mathrm{it}}$$

where,

BPerf=Bank performance (with ROA, ROE, NIM, and TBQ as proxy)
β0=Constant
Β1- β6=Parameter of the explanatory variable represented as:
lnCEOR=CEO remuneration (natural log)
lnCEOT=CEO tenure (natural log)
lnBCREM=Board Chair remuneration (natural log)
lnBCT=Board Chair Tenure (natural log)
lnCSO=CEO Share Ownership (natural log)
lnBGDR=Board Gender (natural log)
μ=disturbance terms
i=8 banks in the sample; and
t=11 time period.

The estimates for the regression are presented in Table 3.

Table 1 presents the descriptive statistics on the managerial incentives measures, and the bank performance measures. The maximum CEO tenure of 19 years was a sunset observation recorded at the inception of the implementation of the revised corporate governance code. The code prescribes a tenure limit of 10 years subject to a maximum of two-terms. The reported maximum value of NGN 201 million as Board Chairman Remuneration is also attributed to the sunset effect prior to the implementation of the revised code of corporate governance in the banking sector. Female representation on bank boards was low at an average of 11.67%, but the rate is comparable to other studies.

Results of the correlation and variance inflation factors in Table 2 show no evidence of multicollinearity.

Following from Table 3, three managerial incentives show a negative significant influence on bank performance in selected deposit money banks in Nigeria: Board Chair remuneration (on ROA, ROE, and TBQ); CEO remuneration (on TBQ and NIM), and CEO Share ownership (on TBQ). Conversely, Board Chair Tenure showed a positive significant effect on bank performance (NIM). These estimates have managerial implications. As banks seek to improve their financial performance, the role of managerial incentives needs to be given a special consideration in order to identify what creates or erodes shareholders׳ value. The dataset provides useful insights for board remuneration and nomination committees, investors, and regulators to understand the influence of managerial incentives in enhancing a deposit money bank׳s performance.
